# Perioperative Management of Rhinoplasty without Epinephrine

**Published:** 2018-05

**Authors:** Franco Bassetto, Vincenzo Vindigni, G Tanzillo

**Affiliations:** Clinic of Plastic, Reconstructive and Aesthetic Surgery, University of Padua, Italy

**Keywords:** Perioperative management, Rhinoplasty, Epinephrine

## Abstract

In the last years, number of rhinoplasty operations is ever increasing, but what can be done when there is a contraindication to use a vasoconstrictor?^2^ In our case, we presented our perioperative management of a female patient, epinephrine intolerant, who was arranged for rhinoplasty.

## INTRODUCTION

In the last years, number of rhinoplasty operations is ever increasing. The nose is highly vascularized and therefore, it is generally recognized effective by using a vasoconstrictor together with a local anaesthetic to perform the procedure.^1^^-^^3^ Providing vasoconstriction and prolonging the anaesthetic effect, are both desirable in this type of surgery. What can we do when there is a contraindication to use a vasoconstrictor?^4^ In our case we present our perioperative management of a female patient, epinephrine intolerant, who was submitted to rhinoplasty.

## CASE REPORT

The patient has been submitted to an open rhinoplasty for the correction of nose tip and bridge, as the type of procedure to be made for the septum. General anaesthesia was managed by demanded doses of sufentanil with an infusion pump to stabilize and maintain hypotension in addition to N2O–O2–sevorane. In the beginning of the operation, the patient received dexamethasone (4 mg) and droperidol (0.6 mg) to prevent postoperative nausea and vomiting (PONV).

We used ropivacaine (10 mg/ml) as local anaesthetic and we placed nose pads soaked with xylomethazoline chlorhydrate (0.1 g) for at least 30 minutes before the begining of the operation. The patient was placed in anti-Trendelenburg position at 30°. During the operation, it has been constantly placed a cold wet gauze on the nose bridge and surgical loupes 4x has been utilized to improve the anatomical visualization and keep the correct surgical plane, with the aim to reduce intraoperative bleeding.

**Fig. 1:**
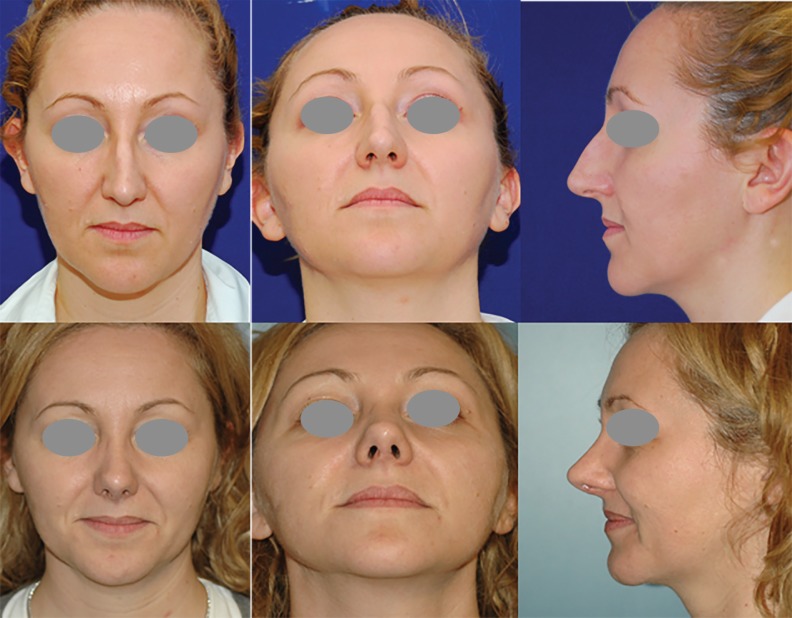
Six months follow up after osteo-cartilaginous hump reduction, low to high osteotomies, cranial resection of alar cartilages and inter- intradomal sutures for nose-tip reshaping

We executed marginal incisions and, after the columellar incision, we cauterized the columellar arteries. Alar cartilages were exposed and nasal vault was undermined. The cartilaginous hump was lowered with angled scissors and nasal bone dorsum was careful rasped with **Joseph rasp** (20 cm length). Lateral osteotomies were performed with a 3 mm osteotome. After we executed cranial resection of alar cartilages, we performed inter- and intradomal sutures for nose-tip reshaping. Finally, we sutured mucosa and columella. The operation lasted 120 minutes, a little more time than that routinely spent for rhinoplasty, regarding the post-operatory follow up (Figure 1). The patient was happy about the result that we obtained, and we were satisfied of the surgery procedure.

## DISCUSSION

The nose is highly vascularized, that explains the regular association of vasoconstrictor and local anesthetic use.^5^ In our case, since we were unable to administer epinephrine, due to a patient intolerance, we had to use some tricks to reduce blood supply. Systemically, anesthesiological management of blood pressure has been of great importance. Remifentanil, in fact, was shown to have hypotensive effects,^6^ this permitted to keep blood pressure constantly as 80/50 mmHg. Locally, we used ropivacaine by virtue of its vasoconstrictor effect,^7^ in addition to its powerful anaesthetic/analgesic effect. We have added decongestant effects induced by xylometazoline chlorhydrate,^8^ the nasal pads that were kept in place for at least 30 minutes.

The patient was placed in anti-Trendelenburg position at 30°, this induced about 15% vascularization decrease in head and neck zone. During the operation, a cold wet gauze has been constantly placed on the nose bridge to keep the vasoconstriction with the same importance surgical carefulness. It was assisted by use of surgical loupes 4x that we could cauterize columellar arteries and mobilize tissues more carefully, identifying avascular surgical plans during rhinoplasty, thus to be traumatic as little as possible. Despite our clinic that routinely uses epinephrine associated to local anesthetic to perform a rhinoplasty, we explained if contraindication to use epinephrine is visible, how it would be possible to perform the same operation, not taking a long time compared to use of epinephrine and without overbleeding.

## CONFLICT OF INTEREST

The authors declare no conflict of interest.
